# pH Control Enables Simultaneous Enhancement of Nitrogen Retention and N_2_O Reduction in *Shewanella loihica* Strain PV-4

**DOI:** 10.3389/fmicb.2017.01820

**Published:** 2017-09-20

**Authors:** Hayeon Kim, Doyoung Park, Sukhwan Yoon

**Affiliations:** Department of Civil and Environmental Engineering, Korea Advanced Institute of Science and Technology Daejeon, South Korea

**Keywords:** denitrification, respiratory ammonification, nitrous oxide (N_2_O), pH, RT-qPCR

## Abstract

pH has been recognized as one of the key environmental parameters with significant impacts on the nitrogen cycle in the environment. In this study, the effects of pH on NO_3_^–^/NO_2_^–^ fate and N_2_O emission were examined with *Shewanella loihica* strain PV-4, an organism with complete denitrification and respiratory ammonification pathways. Strain PV-4 was incubated at varying pH with lactate as the electron donor and NO_3_^–^/NO_2_^–^ and N_2_O as the electron acceptors. When incubated with NO_3_^–^ and N_2_O at pH 6.0, transient accumulation of N_2_O was observed and no significant NH_4_^+^ production was observed. At pH 7.0 and 8.0, strain PV-4 served as a N_2_O sink, as N_2_O concentration decreased consistently without accumulation. Respiratory ammonification was upregulated in the experiments performed at these higher pH values. When NO_2_^–^ was used in place of NO_3_^–^, neither growth nor NO_2_^–^ reduction was observed at pH 6.0. NH_4_^+^ was the exclusive product from NO_2_^–^ reduction at both pH 7.0 and 8.0 and neither production nor consumption of N_2_O was observed, suggesting that NO_2_^–^ regulation superseded pH effects on the nitrogen-oxide dissimilation reactions. When NO_3_^–^ was the electron acceptor, *nirK* transcription was significantly upregulated upon cultivation at pH 6.0, while *nrfA* transcription was significantly upregulated at pH 8.0. The highest level of *nosZ* transcription was observed at pH 6.0 and the lowest at pH 8.0. With NO_2_^–^ as the electron acceptor, transcription profiles of *nirK, nrfA*, and *nosZ* were statistically indistinguishable between pH 7.0 and 8.0. The transcriptions of *nirK* and *nosZ* were severely downregulated regardless of pH. These observations suggested that the kinetic imbalance between N_2_O production and consumption, but neither decrease in expression nor activity of NosZ, was the major cause of N_2_O accumulation at pH 6.0. The findings also suggest that simultaneous enhancement of nitrogen retention and N_2_O emission reduction may be feasible through pH modulation, but only in environments where C:N or NO_2_^–^:NO_3_^–^ ratio does not exhibit overarching control over the NO_3_^–^/NO_2_^–^ reduction pathways.

## Introduction

Nitrous oxide (N_2_O) is a potent greenhouse gas ∼300 times more effective than CO_2_ in causing radiative forcing if present at the same concentration (Lashof and Ahuja, 1990). N_2_O has also been the most powerful ozone depletion agent in the atmosphere since phasing out of chlorofluorocarbons (Portmann et al., 2012). By far, the largest source of N_2_O is biotransformation of reactive nitrogen (e.g., NH_4_^+^, NO_3_^–^, and urea) applied as nitrogen fertilizers to agricultural soils (Ravishankara et al., 2009; Reay et al., 2012). The increase in the atmospheric concentration of N_2_O is strongly correlated to the increase in the global input of nitrogen fertilizers to agricultural soils (Kroeze et al., 1999; Davidson, 2009). Therefore, understanding nitrogen cycling in the environment and developing strategies for sustainable management of soil nitrogen are crucial for efforts to reduce N_2_O emissions.

Limiting soil denitrification by stimulating the reaction that competes with denitrification for the same substrates, NO_3_^–^ or NO_2_^–^, has been proposed as a way to enhance nitrogen retention in agricultural soils (Tiedje et al., 1983; Yoon et al., 2015a). While denitrification is reduction of NO_3_^–^/NO_2_^–^ to N_2_O and N_2_ via NO, respiratory ammonification (also called dissimilatory nitrate/nitrite reduction to ammonium or DNRA) reduces NO_3_^–^/NO_2_^–^ to NH_4_^+^, the form of nitrogen with higher tendency to be retained in pore water or adsorbed to negatively charged particle surfaces (Laima et al., 1999; Silver et al., 2001; Fitzhugh et al., 2003). Recent advances in microbial ecology have identified the environmental parameters that control the competition between denitrification and respiratory ammonification through experiments using axenic cultures or enrichment cultures (Kraft et al., 2014; van den Berg et al., 2015; Yoon et al., 2015a,b). In chemostat experiments with *Shewanella loihica* strain PV-4, electron acceptor limitation due to the high C:N ratio of the feed medium favored the dominance of respiratory ammonification over denitrification (Yoon et al., 2015a). Enrichment of microorganisms capable of DNRA upon electron acceptor limitation was observed in a similar experiment using wastewater as the inoculum (van den Berg et al., 2016). Although contrasting observations were reported, NO_2_^–^:NO_3_^–^ ratios also factored into selection of the NO_3_^–^/NO_2_^–^ reduction pathways in two independent experiments carried out by different research groups (Kraft et al., 2014; Yoon et al., 2015b).

pH is an important environmental parameter with significant impacts on many biogeochemical reactions, and nitrogen-oxide dissimilation is not an exception (Liu et al., 2010, 2014; Dörsch et al., 2012; Qu et al., 2014; Brenzinger et al., 2015; Yoon et al., 2015a). pH was, in fact, suggested as an environmental parameter with significant influence on the fate of NO_3_^–^, based on the experimental results with batch cultures of *S. loihica* strain PV-4 (Yoon et al., 2015a). The adverse effect of acidic pH on N_2_O reduction by nitrous oxide reductases (NosZ) was previously observed in axenic cultures of *Paracoccus denitrificans* and enrichments of soils with varying pH (Bergaust et al., 2010; Liu et al., 2014). In both sets of experiments, acidic pH resulted in transient or permanent accumulations of N_2_O, suggesting that NosZ was less active under acidic conditions. Transcription of *nosZ* genes in these experiments remained intact under acidic pH, suggesting that NosZ inactivation observed at low pH is likely due to post-transcriptional regulation.

In this study, *S. loihica* strain PV-4 was used as a model organism to examine whether upward pH adjustment allows for simultaneous stimulation of respiratory ammonification and N_2_O reduction, and to investigate the relative importance of pH as an effector of nitrogen-oxide dissimilation reactions. The concentrations of the inorganic nitrogen species were monitored in anaerobic batch reactions initially amended with NO_3_^–^/NO_2_^–^ and N_2_O at pH 6.0, 7.0, and 8.0. Transcription profiles of the functional genes encoding dissimilatory NO_2_^–^ reductases (*nirK* and *nrfA*) and N_2_O reductase (*nosZ*) were analyzed with reverse transcription quantitative polymerase chain reaction (RT-qPCR) technique with samples extracted from *S. loihica* strain PV-4 cultures at the varying pH. Limitations do exist in extrapolating experimental results from axenic culture experiments to ecological contexts; nevertheless, these experiments demonstrated that stimulation of respiratory ammonification at high pH conditions shifted the N_2_O-generating NO_3_^–^-reducing organism to a sink of N_2_O. Unlike previous observations, lowering of pH to 6.0 did not lead to inhibition of N_2_O reduction activity in *S. loihica* strain PV-4, suggesting that the cause of N_2_O accumulation was due to the kinetic imbalance of the nitrogen oxide reduction reactions, rather than transcriptional or post-transcriptional regulation. Our observations also suggested that the pH effects on nitrogen-oxide dissimilation reactions were not as influential as the effects of C:N or NO_2_^–^:NO_3_^–^ ratios.

## Materials and Methods

### Media and Culture Conditions

The medium for cultivation of *S. loihica* strain PV-4 was prepared as described previously (Yoon et al., 2013). Medium containing 20 g NaCl, 0.233 g KH_2_PO_4_, 0.46 g K_2_HPO_4_ and 2 mL trace metal solution (Myers and Nealson, 1990) per liter was boiled with N_2_ flushing. pH of the medium was adjusted to 6.0, 7.0, or 8.0 with 5.0 N HCl or 5.0 N NaOH before boiling. After 100-mL aliquots of the degassed medium were distributed to N_2_-flushed 160 mL serum bottles, the bottles were capped with black butyl stoppers (Geo-Microbial Technologies, Inc., Ochelata OK, United States) and aluminum crimp seals and autoclaved. Wolin vitamin solution was added from a filter-sterilized 200X stock solution after autoclaving (Wolin et al., 1964). The medium was amended with sodium lactate, KNO_3_ (or KNO_2_) and NH_4_Cl from sterilized anoxic stock solutions to final concentrations of 560.3 mg/L (5 mM), 101.1 mg/L (1 mM), and 10.7 mg/L (0.2 mM), respectively. The inoculated serum bottles were incubated in dark at 25°C without shaking. After each experiment, final pH was measured to confirm that pH was maintained constant (within ± 0.1 of the initial value).

### Analytical Procedures

Concentrations of NO_3_^–^, NO_2_^–^, NH_4_^+^ and N_2_O were monitored using established analytical procedures. N_2_O concentration was measured using HP 6890 Series gas chromatograph equipped with a HP-PLOT Q column (30 m × 0.320 mm diameter, 20 μm film thickness) and an electron capture detector (Agilent, Palo Alto, CA, United States). Headspace N_2_O concentration was measured by manually injecting 100 μL headspace sample into the gas chromatograph. The detection limit was approximately 2 ppmv. The total amounts of N_2_O in the vessels were calculated from the headspace concentrations using Equation 1.

(1) Total amount of N2O=Va×ChH+Vh×Ch

where C_h_ is the headspace concentration in moles/L, V_a_ and V_h_ are the volumes of the aqueous phase and the headspace, respectively, in liters and H is the dimensionless Henry’s constant of N_2_O. The dimensionless Henry’s constant at 25°C was calculated as previously described (Yoon et al., 2015b). After correcting for the effect of high salt concentration in the medium, the dimensionless Henry’s constant was calculated to be 1.82.

Aqueous NO_2_^–^ and NH_4_^+^ concentrations were determined colorimetrically using HS-NO_2_ (N)-L and HS-NH_3_ (N)-L kits (Humas, Daejeon, Korea), respectively, according to the protocols provided by the manufacturer. Aqueous NO_3_^–^ concentration was determined with Metrohm 863 Basic IC plus ion chromatography system (Metrohm, Riverview, FL, United States) equipped with a Metrosep A Supp 4-250/4.0 anion exchange column. In presentations of the experimental data, the quantities of the nitrogen species were expressed in μmoles/bottle for convenience in mass balance calculations.

### Observation of the pH Effects on Nitrogen-Oxide Dissimilation

Precultures of *S. loihica* strain PV-4 cells were prepared with 5.0 mM lactate and 1.0 mM NO_3_^–^ as the electron donor and the electron acceptor, respectively. These precultures were incubated until NO_3_^–^ and NO_2_^–^ were depleted, and 0.5 mL of the precultures were inoculated to serum bottles with fresh media adjusted to the desired pH (6.0, 7.0, or 8.0) and amended with 5.0 mM lactate and 1 mM NO_3_^–^ or NO_2_^–^. Immediately after inoculation, 0.5 mL of headspace N_2_ was aseptically replaced with >99.999% N_2_O (Samoh Corporation, Daejeon, Korea). With time intervals determined from preliminary experiments, headspace and aqueous phase samples were extracted for monitoring of NO_3_^–^, NO_2_^–^, NH_4_^+^ and N_2_O concentrations and cell densities. For determination of N_2_O concentration, 100 μL of headspace gas was extracted using a 1700-series gastight syringe (Hamilton Company, Reno, NV, United States) and manually injected into the gas chromatograph. Immediately after headspace sampling, 1.5 mL of the aqueous phase was extracted using a disposable 3-mL syringe (Becton Dickinson, Franklin Lakes, NJ, United States). In order to avoid pressure loss in the vessels, 1.6 mL of N_2_ gas was injected before extraction of the liquid samples. After OD_600__nm_ of the extracted cell suspension was measured using Genesys 30 visible spectrophotometer (Thermo Scientific, Waltham, MA, United States), the suspension was centrifuged and the concentrations of NO_3_^–^, NO_2_^–^, NH_4_^+^ and N_2_O in the supernatant were measured. Incubation was carried out until no further change in the concentrations of the nitrogen species was observed.

### RNA Extraction and Analyses of *nrfA, nirK*, and *nosZ* Transcription

The samples for transcription analyses of the genes involved in denitrification and respiratory ammonification were extracted at multiple time points during batch cultivation of *S. loihica* strain PV-4 prepared and carried out identically as described above. For each batch culture amended with NO_3_^–^ and N_2_O, N_2_O concentration was monitored to determine three sampling time points (Supplementary Figure S1). The pH 6.0 cultures were sampled at *t* = 24, 48, and 60 h, the pH 7.0 cultures were sampled at *t* = 11, 24, and 27 h, and pH 8.0 cultures were sampled at *t* = 19, 45, and 52 h. As no growth was observed at pH 6.0 when incubated with NO_2_^–^ and N_2_O, samples were extracted only from the cultures incubated at pH 7.0 and 8.0. These cultures were sampled twice, before the onset of the exponential phase (*t* = 17 and *t* = 20, respectively, for pH 7.0 and pH 8.0 cultures) and during the mid-exponential phase (*t* = 49 h and *t* = 45 h, respectively, for pH 7.0 and pH 8.0 cultures). One-half milliliter samples were collected from three biological replicates upon each sampling event. One milliliter of RNA Protect Bacteria Reagent (Qiagen, Hilden, Germany) was immediately added to each of the aliquots and the mixture was immediately centrifuged for 10 min at 5,000 × *g*. The cell pellets were stored at -80°C until further processing.

An established protocol was used for extraction and purification of total RNA with a few modifications (Park et al., 2017). The cell pellets were thawed in ice and were subjected to disruption with Omni Bead Ruptor 12 Homogenizer (Omni International, Kennesaw, GA, United States) after addition of 350 μL buffer RLT solution and 30 mg of 0.1 mm glass beads (Omni International). Total RNA was extracted using RNeasy Mini Kit (Qiagen) according to the protocol provided by the manufacturer and resulting RNA was eluted with 60 μL RNase-free water. Remaining DNA in the eluent was digested using RNase-free DNase Set Kit (Qiagen) as previously described (Park et al., 2017) and the reaction mix was purified using RNase-free DNase Kit (Qiagen). Reverse transcription was performed with 11 μL of 20 μL eluent using Superscript^TM^ III Reverse Transcriptase (Invitrogen, Carlsbad, CA, United States). The remaining eluent was used later in a quantitative polymerase chain reaction (qPCR) to confirm the absence of contaminating DNA. After reverse transcription, each sample was treated with RNase H (Invitrogen) to remove traces of RNA. The cDNA solution was diluted five-fold with nuclease-free water and stored at -20°C until analyzed using qPCR.

The quantities of *nirK, nrfA, nosZ*, and *recA* genes in the cDNA solutions were determined using the qPCR technique. *nirK* and *nosZ* genes were present in single copies in *S. loihica* strain PV-4 genome and only *nrfA*_0844_ of the two *nrfA*-like genes was targeted, as the expression of *nrfA*_0505_ did not correlate with respiratory ammonification activity (Yoon et al., 2015a). The primers used in this study are listed in **Table 1**. The primers specifically targeting *nrfA, nirK*, and *recA* genes of *S. loihica* strain PV-4 were designed *de novo* using Primer 3 software (Rozen and Skaletsky, 2000) and a previously designed primer was used for amplification of *nosZ* (Yoon et al., 2015a). The target specificities of the primer sets were tested with ordinary PCR and the PCR products were used to construct calibration curves for absolute quantification of target genes. The PCR products were inserted into PCR2.1 vectors and the resulting plasmids were extracted using QIAprep Spin Miniprep Kit (Qiagen). The copy numbers of the extracted plasmids were calculated from the nucleic acid concentration measured with Nanodrop 2000 UV-Vis spectrometer (Thermo Fisher Scientific) and the expected molecular weights of the plasmids. Dilution series of the plasmids ranging from 1 to 10^8^ copies/μL were prepared and used for calibration curve construction. qPCR was performed with QuantStudio^TM^ 3 Real-Time PCR System (Thermo Fisher Scientific) using SYBR Green detection chemistry. 2X Power SYBR Green PCR Master Mix Solution (Applied Biosystems) was used to prepare the reaction mix and a two temperature-cycle procedure was used for qPCR, with 95°C denaturation step (15 s) followed by 60°C elongation step (60 s) in each of 40 cycles. The amplification efficiencies ranged between 94.0 and 99.8% and the *R*^2^ values of the calibration curves were no less than 0.998 (**Table 1**). No amplification was observed in the negative controls without target DNA or cDNA. The qPCR assays for all four target genes were reliable down to 10^1^ copies/μL, and the preparations with 1 copy/μL did not yield consistent results. Consistent melting curves confirmed the specificity of the qPCR reactions.

**Table 1:** Primers used for the RT-qPCR assays.

Primer	Sequence (5′→3′)	Target gene	Amplicon length (bp)	Slope	y-intercept	Amplification efficiency	*R*^2^	Reference
SlonirK755f	TGAGTGAGGTGCTTGAGGTG	*nirK*	237	–3.474	35.552	94.0	0.998	This study
SlonirK991r	TCCAGGTTTCCAGATTGGTC							
SlonrfA724f	CGTCATCCTGAGTTTGAGCA	*nrfA*	227	–3.477	36.396	99.8	0.998	Yoon et al., 2013
SlonrfA950r	TTCTCGGCTATCTGCGACTT							
SlorecA656f	ACGCTTCTGTTCGTCTGGAT	*recA*	245	–3.366	35.931	98.2	0.999	This study
SlorecA900r	GCCAATCTTGTCACCCTT							
SlonosZ599f	ATGGTAAGGAGACGCTGGAA	*nosZ*	160	–3.386	34.713	97.4	0.999	This study
SlonosZ758r	TTGTAGCAGGTAGAGGCGAAG							

The copy numbers of *nirK, nrfA*, and *nosZ* in each cDNA sample were normalized with the copy number of *recA*, the housekeeping gene that encodes DNA recombination/repair protein RecA. Transcription levels of *recA* genes were previously observed to be relatively stable under different growth conditions and growth stages in diverse groups of bacteria including *Streptococcous agalactiae* and *Lactobacillus plantarum* (Marco and Kleerebezem, 2008; Florindo et al., 2012). Thus, *recA* was selected as the most suitable target gene for normalization of the RT-qPCR data to account for the differences in cell densities and overall metabolic activities, as well as mRNA loss during extraction, purification, and reverse transcription procedures. The copy numbers of the *nirK, nrfA*, and *nosZ* genes in the cDNA samples were divided by the copy number of the *recA* gene and the *nirK/recA, nrfA*/*recA*, and *nosZ*/*recA* values were compared across the samples collected from different incubation conditions.

Statistical analyses for the RT-qPCR results were performed using SPSS Statistics 24 software (IBM Corp. Armonk, NY, United States). The RT-qPCR reactions were performed with the samples collected from triplicate reaction vessels and independently processed through extraction, purification and reverse transcription procedures. Statistical analyses were performed with the data transformed to a logarithmic scale.

## Results

### Effects of pH on the NO_3_^–^ Reduction Pathways and N_2_O Fate during NO_3_^–^ Reduction

**FIGURE 1:**
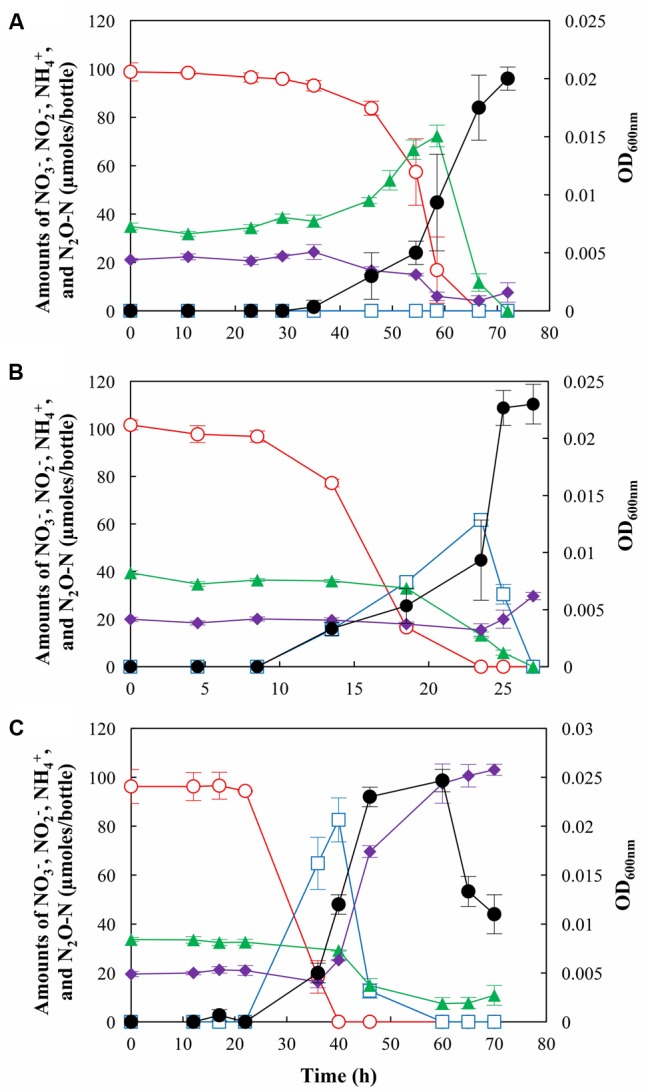
N_2_O production and/or consumption by *Shewanella loihica* strain PV-4 during reduction of NO_3_^–^ at **(A)** pH 6.0, **(B)** pH 7.0, and **(C)** pH 8.0. The amounts of NO_3_^–^ (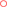), NO_2_^–^ (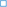), NH_4_^+^ (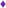), and N_2_O-N (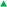) in the culture bottles were monitored until no more change was observed. The OD_600__nm_ values (●) were measured to monitor the cell growth. The data points represent the averages of triplicate cultures and the error bars represent their standard deviations.

pH conditions determined whether *S. loihica* strain PV-4 reduced NO_3_^–^ to NH_4_^+^ (respiratory ammonification) or to N_2_ via N_2_O (through denitrification) and also, whether the batch system functioned as a sink or a source of N_2_O during NO_3_^–^ reduction (**Figure 1**). At all three pH conditions tested, all of NO_3_^–^ added to a nominal concentration of 1.0 mM was consumed after 28 – 72 h after inoculation. The strain PV-4 culture incubated at pH 7.0 had the shortest lag period (∼8 h), while pH 6 and pH 8 cultures both had longer lag phases, as significant decreases in NO_3_^–^ concentrations and increases in the cell densities were observed 35 and 22 h after inoculation, respectively. The maximum observed rates of NO_3_^–^ reduction (pH 6.0: 14.1 μmoles h^-1^ mL^-1^ OD_600__nm_^-1^ at *t* = 54.5 h; pH 7.0: 19.1 μmoles h^-1^mL^-1^ OD_600__nm_^-1^ at *t* = 13.5 h; pH 8.0: 6.4 μmoles h^-1^ mL^-1^ OD_600__nm_^-1^ at *t* = 36 h) and exponential growth rates (0.18, 0.33, and 0.15 h^-1^ at pH 6, 7, and 8, respectively) indicated that neutral pH was optimal for *S. loihica* strain PV-4, but also that overall cellular function of *S. loihica* strain PV-4 was not substantially compromised by the shift of pH within the examined range.

The strain PV-4 cultures incubated at different pH conditions were distinguished by the magnitudes of transient NO_2_^–^accumulation. Accumulation of NO_2_^–^ was not observed in the culture incubated at pH 6.0 and the concentration of NO_2_^–^ remained below the detection limit throughout the experiment, indicating that potential NO_2_^–^ reduction rate was at least as high as the rate of NO_3_^–^ reduction. Contrastingly, at pH 7.0 and 8.0, NO_2_^–^ accumulations up to 61.7 ± 2.6 μmoles/bottle and 82.6 ± 9.0 μmoles/bottle were observed, respectively, indicating that the rates of NO_2_^–^ reduction were slower than the rates of NO_3_^–^ reduction.

At pH 7.0 and 8.0, *S. loihica* strain PV-4 functioned as a net sink of N_2_O, as N_2_O concentrations decreased consistently throughout incubation periods (**Figure 1**). No transient N_2_O accumulation was observed in the cultures incubated at pH 7.0 and 8.0. A clearly distinct trend was observed in the strain PV-4 culture incubated at pH 6.0, as a transient accumulation of N_2_O up to 72.3 ± 4.5 μmoles N_2_O-N/bottle (37.4 μmoles N_2_O-N/bottle higher than initially added N_2_O-N) occurred before NO_3_^–^ depletion. Although N_2_O accumulation was observed, N_2_O reduction was active throughout the experiment. The maximum N_2_O reduction rate of 12.2 μmoles N_2_O-N h^-1^ mL^-1^ OD_600__nm_^-1^ was calculated at *t* = 54.5 h. This rate was comparable to the calculated maximum N_2_O reduction rate of 14.2 μmoles N_2_O-N h^-1^ mL^-1^ OD_600__nm_^-1^ at pH 7.0 at *t* = 23.5 h. At pH 8.0, the maximum observed N_2_O reduction rate (279 nmoles N_2_O-N h^-1^mL^-1^ OD_600__nm_^-1^ at *t* = 40 h) was substantially lower than those observed at pH 6.0 and 7.0. N_2_O reduction stopped after 60 h, when the dissolved N_2_O concentration was lowered to 0.75 μM and no other electron acceptor was available in the medium. Energy gained from N_2_O reduction may not be sufficient *per se* to support growth at the alkaline pH. In fact, no significant growth or N_2_O consumption was observed for 200 h when strain PV-4 was incubated with N_2_O as the sole electron acceptor at pH 8.0 (data not shown).

pH was also a determinant of NO_3_^–^ fate in the strain PV-4 cultures. Nitrogen mass balance was used to estimate the magnitude of denitrification activity, assuming that >90% of NO_3_^–^/NO_2_^–^ was dissimilated to either NH_4_^+^ or denitrification products (Yoon et al., 2015a). At pH 6.0, consumption but not production of NH_4_^+^ was observed, as the amount of NH_4_^+^ decreased from 21.1 ± 0.5 μmoles/bottle to 4.2 ± 2.1 μmoles/bottle after cultivation, indicating that denitrification was the dominant NO_3_^–^ reduction pathway. The respiratory ammonification pathway was mostly switched off, although a statistically insignificant increase (3.4 ± 1.9 μmoles/bottle) in the amount of NH_4_^+^ was observed after *t* = 66.5 h. At pH 7.0, an increase in the NH_4_^+^ concentration was observed between *t* = 25 h and *t* = 27 h and ∼9.6% (9.7 ± 2.3 μmoles) of initially added 101.6 ± 2.1 μmoles NO_3_^–^ was reduced to NH_4_^+^; however, NH_4_^+^ was still a minor product. The distribution of products from NO_3_^–^ reduction at pH 8.0 was distinctively different from the results observed at pH 6.0 and 7.0. NH_4_^+^ was the major product of NO_3_^–^ reduction, as 83.5 ± 1.2 μmoles of NO_3_^–^ was reduced to NH_4_^+^. Respiratory ammonification dominated NO_3_^–^ reduction at the alkaline pH and shutdown of the NirK-mediated NO_2_^–^ reduction activity could be inferred from the mass balance. With diminished denitrification activity, *S. loihica* strain PV-4 cultures functioned as an N_2_O sink at pH 8.0 despite of reduced N_2_O reduction activity.

### Effects of pH on NO_2_^–^ Reduction Pathways and N_2_O Fate during NO_2_^–^ Reduction

**FIGURE 2:**
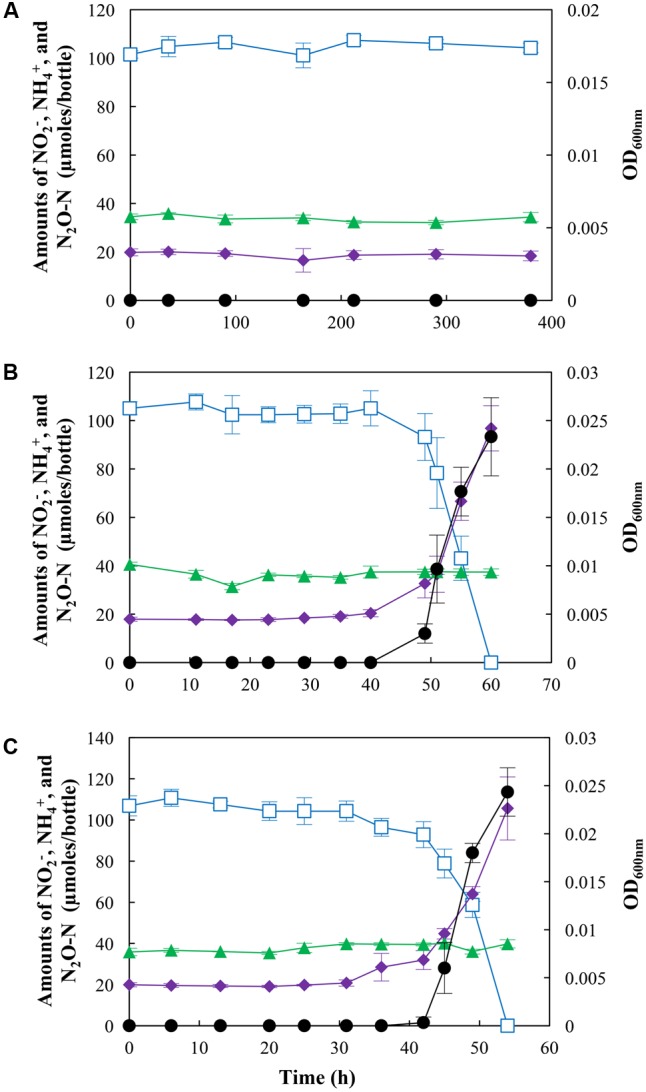
N_2_O production and/or consumption by *Shewanella loihica* strain PV-4 during reduction of NO_2_^–^ at **(A)** pH 6.0, **(B)** pH 7.0, and **(C)** pH 8.0. The amounts of NO_2_^–^ (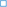), NH_4_^+^ (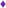), and N_2_O-N (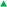) in the culture bottles were monitored until no more change was observed. The OD_600nm_ values (●) were measured to monitor the cell growth. The data points represent the averages of triplicate cultures and the error bars represent their standard deviations.

When *S. loihica* strain PV-4 cultures were amended with NO_2_^–^ instead of NO_3_^–^, the effects of pH on NO_2_^–^ reduction pathways were obscured by the effect of NO_2_^–^ (**Figure 2**). At pH 6.0, neither cell growth or significant change in concentrations of NO_2_^–^, NH_4_^+^, or N_2_O was observed for >300 h. Lowering of the initial NO_2_^–^ concentration to 0.1 mM did not result in cell growth or metabolic activity (data not shown), precluding the possibility that the toxicity of HNO_2_ at the acidic pH was the cause of growth inhibition. The experiments at pH 7.0 and pH 8.0 yielded statistically indistinguishable results (*p* > 0.05), as NH_4_^+^ was the predominant product (78.9 ± 8.5 μmoles and 85.7 ± 5.5 μmoles recovered as NH_4_^+^ at pH 7.0 and 8.0, respectively) regardless of pH. No significant change in the concentration of N_2_O was observed at either pH, indicating that the pathways leading to the production and consumption of N_2_O were inactive when *S. loihica* strain PV-4 was incubated with NO_2_^–^. These results suggested that the pH effect on nitrogen-oxide dissimilation was eclipsed by the NO_2_^–^ effect.

### Effect of pH on Transcription of *nirK, nrfA*, and *nosZ* Genes

**FIGURE 3:**
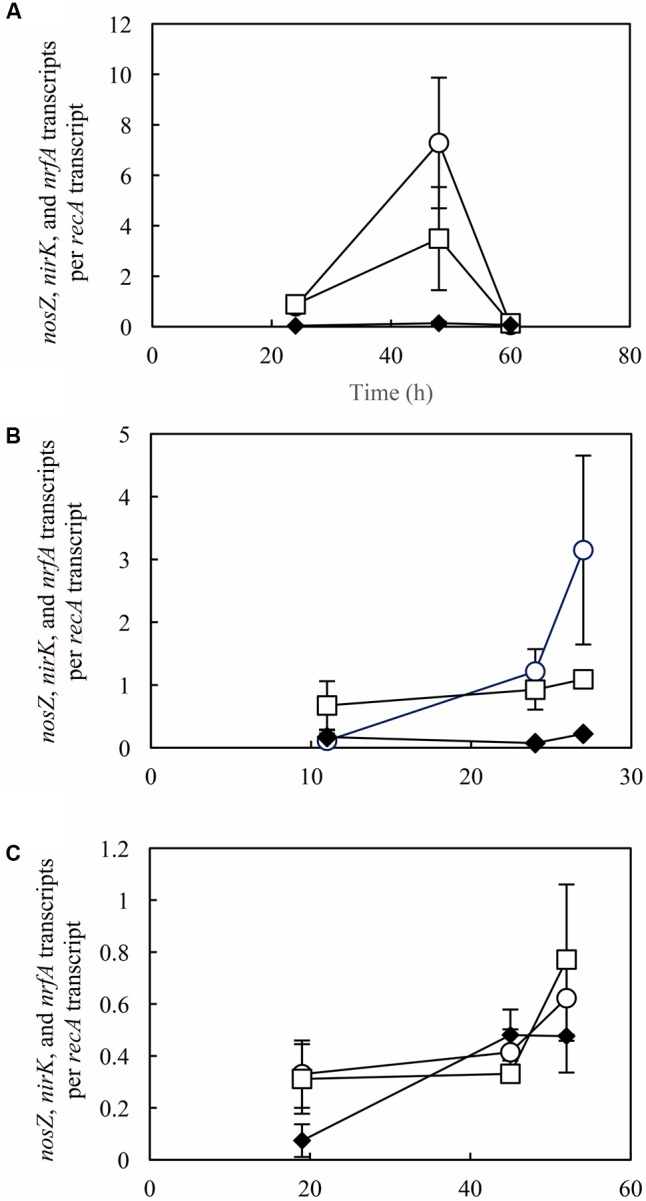
Transcription analyses of *nosZ* (○), *nirK* (□), and *nrfA* (♦) in *Shewanella loihica* strain PV-4 cells grown with NO_3_^–^ and N_2_O at pH **(A)** 6.0, **(B)** 7.0, and **(C)** 8.0. RT-qPCR was performed with samples extracted from batch cultures at the exponential phase. The error bars represent the standard deviations of three biological replicates processed independently through RNA extraction, purification, and reverse transcription procedures.

**FIGURE 4:**
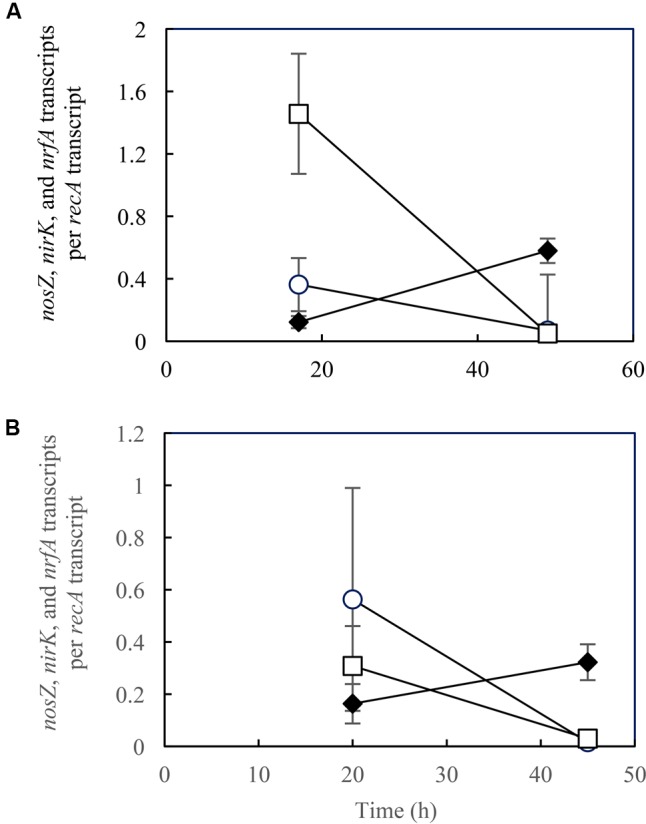
Transcription analyses of *nosZ* (○), *nirK* (□), and *nrfA* (♦) in *Shewanella loihica* strain PV-4 cells grown with NO_2_^–^ and N_2_O at pH **(A)** 7.0, and **(B)** 8.0. RT-qPCR was performed with samples extracted from batch cultures at the exponential phase. The error bars represent the standard deviations of three biological replicates processed independently through RNA extraction, purification, and reverse transcription procedures.

Transcription analyses were performed using RT-qPCR technique with samples extracted at different time points during incubation of *S. loihica* strain PV-4 (**Figures 3, 4**). To account for the differences in the cell densities and overall metabolic activities at different culturing conditions and growth stages, the *nosZ* transcription data were normalized with the *recA* transcription data. At pH 6.0 and pH 7.0, *nirK* transcription levels were at least five-fold higher than those of *nrfA* throughout the incubation periods except at *t* = 60 h at pH 6.0. The reduced transcription of *nirK* at this time point may be due to the depletion of NO_3_^–^. At pH 8.0, the differences in transcription of *nrfA* and *nirK* were insignificant (*p* > 0.05) throughout incubation (**Figure 3**). These transcription profiles explained the dominance of denitrification activity at pH 6.0 and 7.0 and the predominance of respiratory ammonification activity at pH 8.0. As expected from the sustained N_2_O reduction activity at pH 6.0, *nosZ* transcription was not adversely affected by the acidic pH (**Figure 3**). Transcription of *nosZ* was significantly more active at pH 6.0 and 7.0 than at pH 8.0, as the maximum *nosZ* transcripts / *recA* transcript values of 7.28 ± 2.59, 3.15 ± 1.50, and 0.62 ± 0.16 were recovered from samples extracted from the mid-exponential-phase cultures incubated at pH 6.0 (at *t* = 48 h), 7.0 (at *t* = 27 h), and 8.0 (at *t* = 52 h), respectively.

In the samples extracted from *S. loihica* strain PV-4 cultures grown with NO_2_^–^ and N_2_O as the electron acceptors, distinguishable shifts in *nirK, nrfA*, and *nosZ* transcription levels from the lag phase (*t* = 17 h for pH 7.0 and *t* = 20 h for pH 8.0) to the mid-exponential phase (i.e., at *t* = 49 h for pH 7.0 and *t* = 45 h for pH 8.0) were observed (**Figure 4**). At the mid-exponential phase when active NO_3_^–^/NO_2_^–^ reduction occurred, *nrfA* transcription levels were an order of magnitude higher than *nirK* transcription levels. The *nirK* transcription-to-*nrfA* transcription ratios were statistically indistinguishable between pH 7.0 and pH 8.0 (*p* > 0.05). These observations were in agreement with the predominance of respiratory ammonification. The diminished transcription of the genes responsible for N_2_O-producing reactions (*nirK*) and N_2_O-consuming reaction (*nosZ*) explained the absence of N_2_O production or consumption in the cultures amended with NO_2_^–^ and N_2_O.

## Discussion

The experiments performed with *S. loihica* strain PV-4 with NO_3_^–^ and N_2_O as electron acceptors confirmed the previous finding that pH is a significant environmental parameter that regulate nitrogen-oxide dissimilation reactions. pH was previously suggested as one of the environmental parameters that determine the fate of NO_3_^–^ in axenic cultures of *S. loihica* strain PV-4 and also, in complex mixed cultures (Stevens et al., 1998; Yoon et al., 2015a). The endpoint measurements of nitrogen species suggested enhancements of respiratory ammonification activity (or DNRA in ecological context) under alkaline conditions; however, no experiment has been performed to monitor the time-dependent progression of the reactions nor to investigate the molecular basis of this regulation. The findings from the RT-qPCR assays in this study confirmed that the transcription regulation of *nirK* and *nrfA* was the cause of bifurcation of NO_3_^–^ fate at different pH. This finding was consistent with the previous observations that the response of *S. loihica* strain PV-4 to shifting C:N ratio and NO_2_^–^:NO_3_^–^ ratio occurred at the transcription level (Yoon et al., 2015a,b), suggesting that the organism actively selects for the NO_3_^–^/NO_2_^–^ reduction pathway that ensures the most efficient use of the electron acceptors in response to the shifting growth conditions. In the cases of C:N ratio and NO_2_^–^:NO_3_^–^ ratio, the rationale for such pathway selection was explained as the selection for more favorable energetics and higher electron transfer efficiency (Yoon et al., 2015a,b). The rationale for pathway selection upon pH shift may be found from the activities of the nitrite reductases at different pH. The optimal activities of isolated CuNIR were observed at pH < 7.0 (Abraham et al., 1997; Jacobson et al., 2007), while isolated NrfA proteins had pH optima at pH > 7.5 without exception (Liu and Peck, 1981; Kajie and Anraku, 1986). *S. loihica* strain PV-4 may have evolved to increase the expression of the nitrite reductase that has higher activity at the pH of its environ.

The absence of growth with NO_2_^–^ and N_2_O as the electron acceptors at pH 6.0 was an unanticipated result. NO_2_^–^ and its protonated form HNO_2_ are widely known to be toxic to microorganisms. *Shewanella loihica* strain PV-4 was previously found to be vulnerable to the elevated NO_2_^–^ concentrations (>2.0 mM NO_2_^–^); however, toxicity of NO_2_^–^ at 1.0 mM concentration was not sufficient to have an adverse impact on cell growth (Yoon et al., 2015b). Nitrous acid (HNO_2_) is known to be more toxic than NO_2_^–^ (Jiang et al., 2011). As HNO_2_/NO_2_^–^ couple has a pK_a_ value of 3.15, the concentration of HNO_2_ would be ∼10-fold higher at pH 6.0 than at pH 7.0, provided that the total HNO_2_/NO_2_^–^ concentration remains unchanged. If increased HNO_2_ toxicity was the reason for the lack of growth, strain PV-4 would have grown with lowered NO_2_^–^ concentration (0.1 mM). Thus, the absence of growth with 0.1 mM NO_2_^–^ suggested that the HNO_2_ toxicity was not the cause for the growth inhibition at pH 6.0. Although largely speculative, the absence of growth may be explained with the differential transcription levels and activities of the enzymes involved with nitrogen-oxide dissimilation at the varying pH conditions. The elevated NO_2_^–^:NO_3_^–^ ratio could have resulted in down-regulation of *nirK* and *nosZ* transcription and up-regulation of *nrfA* transcription. As NrfA has diminished activity at low pH, *S. loihica* strain PV-4 may lack active NO_2_^–^-reducing enzymes and thus, may not be able to generate sufficient energy for growth.

The transient N_2_O accumulation observed during NO_3_^–^ reduction by *S. loihica* strain PV-4 was consistent with the observations made previously with *P. denitrificans* and soil microbial consortia (Liu et al., 2010, 2014; Bergaust et al., 2012; Brenzinger et al., 2015). N_2_O peak was observed only at the lowest pH tested, pH 6.0, while N_2_O was steadily reduced at higher pH. The accumulation of N_2_O in the *S. loihica* strain PV-4 cultures at pH 6.0 was not accompanied with the decrease in transcription of *nosZ* or diminished N_2_O reduction activity, precluding transcriptional or post-transcriptional regulation of NosZ as the cause of N_2_O accumulation (Liu et al., 2014). Instead, the findings in this study suggest another mechanism that may contribute to accumulation of N_2_O in acidic environments. The transcription of *nirK*, the gene that encodes for the copper-dependent nitrite reductase, was significantly up-regulated at pH 6.0 as compared to the other pH conditions. Rapid NO_2_^–^ reduction accompanied the enhanced *nirK* transcription. The N_2_O reduction rates were, in fact, higher at pH 6.0 than at pH 8.0; however, the rate of N_2_O production from NO_2_^–^ was higher than the N_2_O consumption rate at the acidic pH. Such kinetic imbalance in the chain of reactions constituting the denitrification pathway in strain PV-4 may be the major cause of N_2_O accumulation at pH 6.0. Upregulation of transcription of NO-forming nitrite reductase genes (i.e., *nirK* or *nirS*) under acidic pH was previously observed upon incubation of soil inoculum (Liu et al., 2014). Our observations suggest that the kinetic imbalance caused by enhancement of N_2_O-producing reaction (i.e., NO_2_^–^ reduction) as relative to N_2_O-removal reaction (i.e., N_2_O reduction) may be one of the major cause of enhanced N_2_O emissions from moderately acidic environments.

The positive correlation of DNRA activity with pH has been observed in experiments with soils and sediments (Stevens et al., 1998; Zhang et al., 2015), although other experimental results showed dominance of denitrification as the NO_3_^–^/NO_2_^–^ reduction pathway even under alkaline conditions (Liu et al., 2010, 2014). Regulation of denitrification and DNRA activity in *S. loihica* strain PV-4 was previously observed to be hierarchical, as the effect of C:N ratio overshadowed the effect of NO_2_^–^:NO_3_^–^ ratio when either lactate or NO_3_^–^ was limiting (Yoon et al., 2015b). Likewise, the effect of pH was eclipsed by the NO_2_^–^:NO_3_^–^ effect in this study, as NO_2_^–^ was reduced exclusively via respiratory ammonification pathway regardless of pH. This hierarchical regulation may be applicable to soils and sediments and pH may be a determinant of the fate of NO_3_^–^ and NO_2_^–^ only when their reduction pathway is not predetermined by overarching environmental factors. Agricultural soils simultaneously exhibiting both denitrification and DNRA activities are not rare (Burgin and Hamilton, 2007). pH control may be essential in management of these ‘ambivalent’ agricultural soils, as maintenance of alkaline conditions would reduce nitrogen loss while shifting the soils toward sink of N_2_O. Pure culture experiments may not be sufficient to portray the complex nature of environmental systems; however, the observations with *S. loihica* strain PV-4 certainly demonstrate the feasibility of manipulation of soil nitrogen cycling to simultaneously reduce N_2_O emission and promote N retention via pH control.

## Author Contributions

HK performed the experiments and analyzed data. HK and DP developed the experimental methodology for RT-qPCR assays. SY planned the research and designed the experiments. HK and SY wrote the manuscript. All authors discussed results and commented on the manuscript.

## Conflict of Interest Statement

The authors declare that the research was conducted in the absence of any commercial or financial relationships that could be construed as a potential conflict of interest.
