# Supplementary material for: pH Control Enables Simultaneous Enhancement of Nitrogen Retention and N2O Reduction in Shewanella loihica Strain PV-4
Source: Front Microbiol. 2017 Sep 20;8:1820. doi: 10.3389/fmicb.2017.01820 (PMC5611402; doi:10.3389/fmicb.2017.01820)
Supplement: Supplementary file 1 [file Data_Sheet_1.DOCX]

***Supplementary Material***

**pH control enables simultaneous enhancement of nitrogen retention and N_2_O reduction in *Shewanella loihica* strain PV-4**

**Hayeon Kim^1^, Doyoung Park^1^ and Sukhwan Yoon^1,*^**

^1^Department of Civil and Environmental Engineering, KAIST, Daejeon, Korea

* Correspondence: Sukhwan Yoon, [syoon80@kaist.ac.kr](mailto:syoon80@kaist.ac.kr)

Supplementary Figure

**Figure S1.** N_2_O monitoring for sampling of biomass for transcription analyses. The amounts of N_2_O in the culture bottles incubated at pH 6.0 (○), 7.0 (◊) and 8.0 (△) were monitored and exponential phases were determined based on the N_2_O consumption curves. The arrow marks indicate the sampling time points
